# Quantitative omics and its application to study virus-host interactions—a new frontier

**DOI:** 10.3389/fmicb.2013.00031

**Published:** 2013-03-05

**Authors:** Ben Berkhout, Kevin M. Coombs

**Affiliations:** ^1^Department of Medical Microbiology, Laboratory of Experimental Virology, University of AmsterdamAmsterdam, Netherlands; ^2^Department of Medical Microbiology, Manitoba Centre for Proteomics and Systems Biology, University of ManitobaWinnipeg, MB, Canada

Work during the past few decades has provided a good understanding of alterations that occur in viruses when they interact with host cells. This is generally possible because of the relative simplicity of virus systems, as compared to the more complex cellular systems. There has also been advancement made in delineating host genomic responses to virus infection because of the availability of gene arrays for diverse organisms. However, work that seeks to equally comprehend host responses to virus infection at the protein level (proteomics) has lagged behind because of technical challenges and the great complexity of the cellular proteome.

This series consists of six review articles, three original research papers and a single commentary that all focus on current research activities in the field of proteomics of virus-infected host cells, all designed to begin to fill these important gaps in our knowledge. The articles span quantitative proteomic analyses of host protein alterations induced upon virus infection of a range of host cells: archaeal, insect, and various animal systems. Several articles present results and reviews of new discovery-based functional assays to assess broad enzymatic groups within these experimental systems to complement the quantitative proteomics assays.

The review article by Enrique Santamaria and co-workers provides an overview of mass spectrometry (MS)-based proteomics and how this method has been useful for delineating some of the interactions between herpes simplex virus type 1 (HSV-1) and the host cells (Santamaria et al., 2012). HSV-1 has a unique and intimate relationship with its host, being capable of inducing either a lytic infection cycle or becoming dormant by establishment of the latent state, from which it can be reactivated at later times. As a relatively large and complex particle, HSV-1 virions would be expected to have myriad interactions with host cells. This article provides a broad overview of many of the strategies being used to elucidate proteomic profiling and interactome networks.

The review article by Lippé also presents work aimed at deciphering interactions between HSV and the host cells (Lippe, 2012). In particular, the focus is on specific host cell proteins that are packaged in virion particles and that may be important for viral replication. There is accumulating evidence that a variety of viruses package specific host cell proteins to modulate subsequent virus replication steps by interacting with components of the host cell or organism.

The review by Kramer and colleagues provides an update on another medically relevant human pathogen, the human immunodeficiency virus type 1 (HIV-1), with an emphasis on interactions between the virus and T cells (Kramer et al., 2012). A variety of current challenges and limitations in peptide and protein identification are highlighted, along with the description of new approaches to more rapidly identify potential targets with improved algorithms and by making use of label-free quantitative methodologies. Several authors within this volume highlight the need to couple these discovery-based methods with follow-up experimental validation, including the confirmation of biological relevance of positive hits via knockdown screens.

The review article by Shahiduzzaman and Coombs explores a relatively new area of proteomics and the application of activity-based protein profiling (ABPP) to study virus-host interactions (Shahiduzzaman and Coombs, 2012). ABPP has been used in several non-viral systems to identify and quantify the levels of different classes of enzymes. Thus, this approach offers a complementary set of analyses to those that examine the overall differential protein quantities. ABPP allows the assessment of relative functional activities of all enzymes within each of various broad classes.

The review by Monteiro and co-workers discusses some of the applications of proteomic analyses of baculoviruses (Monteiro et al., 2012). These viruses infect insects and have become a popular tool for the expression of eukaryotic proteins. Baculoviruses are amongst the more complex viral systems with large genomes. It is argued that the study of these viruses as well as the analysis of virus-host cell interactions is hampered by this complexity and the lack of genomic information on the host side. Nevertheless, advances are being made on several fronts to understand the complex interactions between this virus and the host.

Most of the original research and review articles presented above focus on individual virus-host model systems. The final review article by Zheng and colleagues provides a holistic overview of current approaches being undertaken to better define and functionally validate responses elicited within the host proteome by virus infection (Zheng et al., 2012). This includes a comprehensive overview of most of the quantitative approaches being undertaken and several of the functional validations being employed by the growing number of researchers in these areas.

In the first of three original research papers within this volume, Jiang and colleagues label HeLa cells with either normal isotopic amino acids or heavy forms of arginine and lysine in the SILAC approach (Jiang et al., 2012). They infect non-labeled cells with a mammalian reovirus and compare the identities of proteins detected either without enrichment or after non-selective enrichment with Proteominer™ beads. These authors quantitatively compared host protein alterations and identified several thousand of such modifications by comparing the two situations (± enrichment). The proteins and cellular pathways that were either up- or down-regulated were identified. The authors conclude that enrichment does not provide a deeper mining of the host proteome. An accompanying commentary by Speijer points out some of the inherent assumptions and potential limitations of such a study (Speijer, 2012).

The research article by Milev and co-workers describes characterization of Staufen 1, a host protein that binds double-stranded RNA and that is associated with HIV-derived ribonucleoprotein complexes (Milev et al., 2012). This study uses tandem affinity approaches to identify more than 200 host proteins that interact with this viral complex and defines alterations induced in the complex by Staufen 1. The role of many of the identified host proteins is subsequently validated and the intracellular distribution is also determined. This provides a much more comprehensive picture of these important molecules and their intracellular interactions, which may pave the way toward the development of new therapeutic approaches.

The final article by Maaty and colleagues provides a global analysis of host responses to virus infection in an archaeal system (Maaty et al., 2012). Major potential benefits of this “primitive” experimental system relate to its unique placement in evolution. This study employs a multi-faceted omics set of approaches to delineate the fate of host cell RNA and protein after virus infection, both with respect to quantitative aspects and enzymatic activities. This study greatly advances a previously underappreciated area. Perhaps more importantly, comparison of the protein and functional activities that are altered by archaeal viruses to the quantitative and functional perturbations induced by “higher” animal viruses may eventually provide the evolutionary framework for a better understanding of the virus-host interaction in terms of conserved and divergent host genes and proteins.

In summary, these articles present overviews and ongoing original research in one of the “frontier” areas of virology—a better understanding of the complexity of various host omic responses to virus infection, with some examples of proteomic and functional genomic approaches.
